# Inhibitory Effect of Baicalin and Baicalein on Ovarian Cancer Cells

**DOI:** 10.3390/ijms14036012

**Published:** 2013-03-15

**Authors:** Jianchu Chen, Zhaoliang Li, Allen Y. Chen, Xingqian Ye, Haitao Luo, Gary O. Rankin, Yi Charlie Chen

**Affiliations:** 1 Food Science and Nutrition Department, Zhejiang University, Hangzhou 310029, China; E-Mails: jc@zju.edu.cn (J.C.); psu@zju.edu.cn (X.Y.); 2 Natural Science Division, Alderson-Broaddus College, Philippi, WV 26416, USA; E-Mails: liz@ab.edu (Z.L.); luoh@ab.edu (H.L.); 3 Department of Pharmaceutical Sciences, West Virginia University, Morgantown, WV 26506, USA; E-Mail: al50chen@gmail.com; 4 Department of Pharmacology, Physiology and Toxicology, Joan C. Edwards School of Medicine, Marshall University, Huntington, WV 25755, USA; E-Mail: rankin@marshall.edu

**Keywords:** ovarian cancer, baicalin, baicalein, cell viability, VEGF, HIF-1α

## Abstract

Ovarian cancer is one of the primary causes of death for women all through the Western world. Baicalin and baicalein are naturally occurring flavonoids that are found in the roots and leaves of some Chinese medicinal plants and are thought to have antioxidant activity and possible anti-angiogenic, anti-cancer, anxiolytic, anti-inflammatory and neuroprotective activities. Two kinds of ovarian cancer (OVCAR-3 and CP-70) cell lines and a normal ovarian cell line (IOSE-364) were selected to be investigated in the inhibitory effect of baicalin and baicalein on cancer cells. Largely, baicalin and baicalein inhibited ovarian cancer cell viability in both ovarian cancer cell lines with LD_50_ values in the range of 45–55 μM for baicalin and 25–40 μM for baicalein. On the other hand, both compounds had fewer inhibitory effects on normal ovarian cells viability with LD_50_ values of 177 μM for baicalin and 68 μM for baicalein. Baicalin decreased expression of VEGF (20 μM), cMyc (80 μM), and NFkB (20 μM); baicalein decreased expression of VEGF (10 μM), HIF-1α (20 μM), cMyc (20 μM), and NFkB (40 μM). Therefore baicalein is more effective in inhibiting cancer cell viability and expression of VEGF, HIF-1α, cMyc, and NFκB in both ovarian cancer cell lines. It seems that baicalein inhibited cancer cell viability through the inhibition of cancer promoting genes expression including VEGF, HIF-1α, cMyc, and NFκB. Overall, this study showed that baicalein and baicalin significantly inhibited the viability of ovarian cancer cells, while generally exerting less of an effect on normal cells. They have potential for chemoprevention and treatment of ovarian cancers.

## 1. Introduction

Ovarian cancer is one of the most important malignancies for women in the world, ranking as the fifth leading cause of cancer-related deaths [1]. Due to a lack of effective biomarkers for screening [2,3], nearly 60%–70% of ovarian cancers are diagnosed at advanced stages [4], with a poor prognosis of about 30% for a 5-year survival rate [5]. This situation is discouraging but also provides plenty of opportunities for chemoprevention of early-stage ovarian cancers in women. While the search for better biomarkers continues, the strategy of chemoprevention with dietary compounds appears attractive for the vast majority of healthy women who are more or less at a risk of developing ovarian cancer.

Vascular Endothelial Growth Factor (VEGF), initially named vascular permeability factor (VPF), is an important regulator of angiogenesis and vasculogenesis [6]. Angiogenesis occurs in normal processes related to the female reproductive cycle and in pathological processes such as tumor growth and metastasis, diabetic retinopathy, rheumatoid arthritis, or after tissue ischemia [7]. Vasculogenesis involves the formation of blood vessels through the differentiation of endothelial cells from mesodermal precursors. Whereas vasculogenesis is restricted to embryonic development, angiogenesis operates throughout life when new vascularization is required [8]. VEGF gene expression is controlled by oxygen tension, growth factors, hormones, and oncogenes [9]. Hypoxia induces VEGF expression through hypoxia-inducible factor 1 (HIF-1), which is composed of HIF-1α and HIF-1β subunits, with the former one being inducible [10]. Proto-oncogene c-Myc enforces cellular proliferation and growth in tumors [11] and cooperates with HIF-1 in inducing VEGF expression [10].

Flavonoids are natural polyphenols found in many different foods especially in fruits and vegetables [6,12]. The interaction that flavonoids have with antioxidants allows for the neutralization of the effects of reactive substances called free radicals, which damage cells and therefore lead to disease [7,13]. Research has shown that dietary flavonoids decrease the risk of a variety of health problems like cardiovascular disease [9], prostate cancer [11], colorectal cancer [12], ovarian cancer [14], and renal cancer [6] in humans. Additionally, flavonoids help to encourage the inhibition of growth and propagation [1], inhibition of VEGF and HIF-1 [14,15], NFkB and cMyc [16] expression, and prompt cell toxicity [17,18] in cancer cells.

Baicalein is a polyphenolic substance and a member of the flavone subclass of flavonoids. We hypothesize that baicalin and baicalein inhibit cell viability and genes involved in cancer growth in ovarian cancer cells. Baicalein’s conjugate, baicalein-7-glucuronide, also called baicalin, is found naturally, mainly in the roots of the Baical skullcap (*Scutellaria baicalensis Georgi*). It is also found in the leaves of the commonly used herb thyme (*Thymus vulgaris*), the mad-dog skullcap (*Scutellaria lateriflora L*.), and the midnight horror or broken bones plant (*Oroxylum indicum*) [15,19].

However, there is no report about the effects of baicalin and baicalein on ovarian cancer. In this article, two kinds of ovarian cancer (OVCAR-3 and CP-70) cell lines and a normal ovarian cell line (IOSE-364) were chosen and treated with baicalin and baicalein at different concentrations to test their inhibitory effects on viability, phosphorylation of Akt, and expression of several genes including VEGF, HIF-1α, cMyc, NFκB, and PTEN. This study hopes to provide new insights into the mechanisms and potential for chemoprevention of baicalin and baicalein.

## 2. Results and Discussion

### 2.1. Effect of Baicalin and Baicalein on Ovarian Cancer Cells Viability

As women age, the risk of developing ovarian cancer increases [20]. Little research has been conducted to correlate lifestyle and ovarian cancer risk, although diets high in saturated fat and low in vegetables have been linked to ovarian cancer multiple times [21]. Fruits, vegetables, and certain plants have an abundance of flavonoids as their main ingredient, and it is believed that flavonoids play an important part in the anticarcinogenic properties of plants through their antioxidant, antiestrogenic, antiproliferative, antiangiogenic, and anti-inflammatory elements [22]. Baicalin and baicalein are flavonoids that show a strong possibility for decreasing the incidence of cancer, but further studies are required detailing their effects and mechanism of action in ovarian cancers [23].

**Figure 1:**
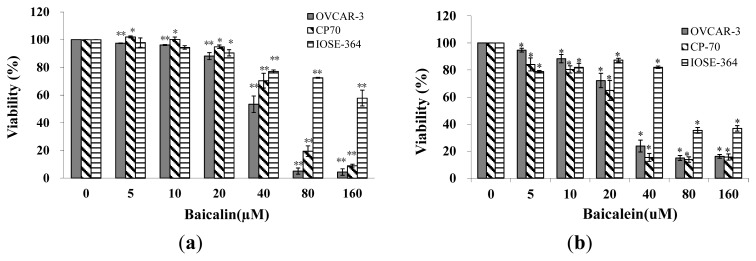
Effect of baicalin and baicalein on viability of OVCAR-3, CP70 and IOSE-364. Cells were treated with different concentrations of (**a**) baicalin or (**b**) baicalein for 24 h, cell viability was colorimetrically determined by a MTS-based method and expressed as percentages of control. Values are expressed as mean ± SD, ** *p* < 0.01, * *p* < 0.05 *vs.* control.

Ovarian cancer cells (OVCAR-3 and A2780/CP70) and normal ovarian cell (IOSE-364) were treated with baicalin and baicalein for 24 h and assayed for cell viability. As shown in Figure 1a,b, an overall inhibitory effect of baicalin and baicalein on ovarian cancer cell (OVCAR-3 and A2780/CP-70) viability was observed. The inhibitory effect increased significantly when their concentrations were more than 40-μM. OVCAR-3 cell viability was inhibited to 53%, 5%, 4%, and CP-70 cell viability was inhibited to 70%, 19%, 9% by 40-μM, 80-μM, 160-μM baicalin treatment, respectively. Similarly, OVCAR-3 cell viability was inhibited to 24%, 15%, 16%, and CP-70 cell viability was inhibited to 15%, 14%, 16% by 40-μM, 80-μM, 160-μM baicalein treatment respectively. LD50 were calculated for two cancer cell lines and the normal ovarian cell line: 44.6 μM for OVCAR-3, 55.2 μM for A2780/CP70, and 177.0 μM for IOSE-364 treated by baicalin, 39.4 μM for OVCAR-3, 24.3 μM for A2780/CP70, and 68.0 μM for IOSE-364 treated by baicalein. The inhibitory effects of baicalin and baicalein on normal ovarian cells (IOSE-364) were less than on ovarian cancer cells. Therefore, baicalin and baicalein selectively inhibit cancer cells while having significantly fewer inhibitory effects on normal ovarian cells. Baicalein is more effective in inhibiting ovarian cancer and normal cells than baicalin.

Recently, traditional Chinese medicines have been recognized as a new basis of anticancer drugs, able to enhance the effectiveness of chemotherapy and to reduce the side effects of cancer treatment. However, the healing mechanisms are still currently unknown [24]. Baicalin’s and baicalein’s effects in lowering ovarian cancer risk cannot be explained solely by the inhibitory properties explored in this study. This experiment showed that baicalin and baicalein significantly inhibit cancer cell viability, while simultaneously distinguishing between tumor cells and normal cells, a property shared by only a few chemicals. The lessened toxicity of these two compounds on normal ovarian cells will help in reducing the toxic side effects of the cancer treatment. Moreover, in both *in vitro* and animal studies, baicalein has shown sufficient anti-inflammatory and antioxidant effects to suggest that this substance might have extensive cytoprotective properties that could be useful in a broad spectrum of diseases including cancer, heart disease, diabetes, stroke, neurodegenerative disorders, and chronic inflammatory diseases, among others [25,26]. Baicalin inhibited the proliferation of human breast cancer cells [19] and also HUVEC cells [27], highlighting its potential as an anticancer agent.

### 2.2. Effect of Baicalin and Baicalein on VEGF Protein Expression in OVCAR-3, CP70 and IOSE-364 Cells

**Figure 2:**
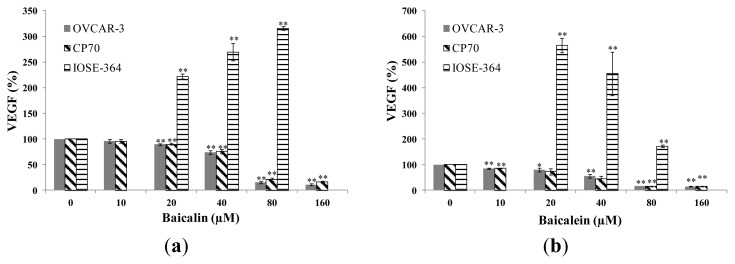
Effect of bacalin and baicalein on VEGF protein expression in OVCAR-3, CP70 and IOSE-364 cells. Cells were treated with different concentrations of (**a**) baicalin or (**b**) baicalein for 24 h, the VEGF expression levels were determined by ELISA analysis and expressed as percentage of control. Values are expressed as mean ± SD, ** *p* < 0.01, * *p* < 0.05 *vs.* control.

Figure 2a,b show that baicalin and baicalein significantly inhibited the expression of VEGF in both ovarian cancer cells, and the inhibitory effect increased with increasing treatment concentrations. On the other hand, baicalin and baicalein did not inhibit the expression of VEGF in the normal ovarian cells IOSE-364. Actually, our results showed that both compounds had increased VEGF expression in the normal cells. An increased VEGF expression in normal cells will increase the expression of cell cycle related proteins that promote transition from G1 phase to the S phase, thus result in the increased survival of the normal cells [27]. VEGF protein levels were inhibited to 72%, 7%, 3% in OVCAR-3 cell and 73%, 13%, 8% in CP70 cell by 40-μM, 80-μM, 160-μM baicalin treatment. Likewise, VEGF protein level were inhibited to 52%, 9%, 7% in OVCAR-3 cell and 46%, 8%, 7% in CP70 cell by 40-μM, 80-μM, 160-μM baicalein treatment, respectively. For the IOSE-364 cells, VEGF protein levels were increased to 222%, 270%, 316% by 20-μM, 40-μM, 80-μM baicalin treatment and 565%, 455%, 173% by 20-μM, 40-μM, 80-μM baicalein treatment. Baicalein is more effective in inhibiting VEGF expression than baicalin in ovarian cancer cells. Although our results about baicalin’s effect on VEGF expression contradict the report by Zhang et al’s finding [28], our results on baicalein agrees with the finding by Ling *et al*. [27] that Baicalein was found to inhibit cell viability through VEGF expression in HUVEC cells.

### 2.3. Effect of Baicalin and Baicalein on HIF-1α Protein and mRNA Expression in OVCAR-3, CP70, and IOSE-364 Cells

**Figure 3:**
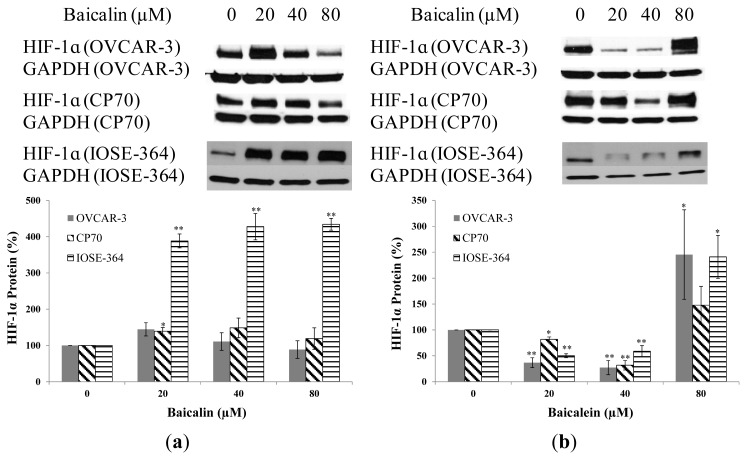
Effect of baicalin and baicalein on HIF-1α protein expression in OVCAR-3, CP70, and IOSE-364 cells. Cells were treated with different concentrations of (**a**) baicalin or (**b**) baicalein for 24 h, the HIF-1α expression levels were determined by Western Blot analysis. GAPDH was used as a control to ensure an equal amount of loaded protein. Values are expressed as mean ± SD, **p < 0.01, * *p* < 0.05 *vs.* control.

Baicalin and baicalein had different effects on HIF-1α protein expression in ovarian cancer cells. Baicalin slightly promoted HIF-1α expression in both ovarian cancer cells, while HIF-1α expression increased rapidly to 388%, 428%, 434% by 20-μM, 40-μM, 80-μM baicalin treatment respectively in normal ovarian cells IOSE-364 (Figure 3a). Baicalein significantly inhibited HIF-1α expression among OVCAR-3, CP70 and IOSE-364 cells at concentrations of 20-μM and 40-μM. But, when treatment concentration was 80-μM, HIF-1α expression increased rapidly to 244% in OVCAR-3 cells, 148% in CP70 cells and 241% in IOSE-364 cells (Figure 3b). Again, both compounds showed no inhibitory effects on normal cells; actually they increased HIF-1α expression which might offset their beneficial effect on cancer treatment. Further studies are warranted to study the mechanism of HIF-1α activation in normal cells. It was found baicalein inhibited cell viability through inhibition of HIF-1α expression in murine microglial cells [29].

**Figure 4:**
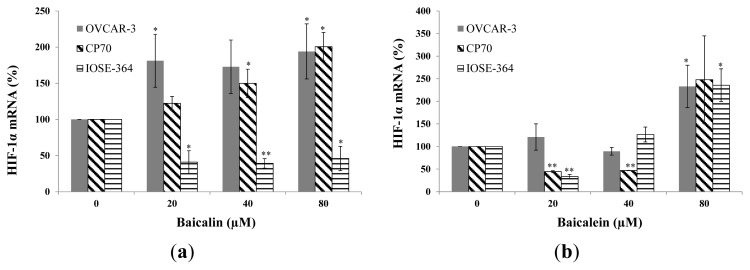
Effect of baicalin and baicalein on HIF-1α mRNA expression in OVCAR-3, CP70, and IOSE364 cells. Cells were treated with different concentrations of (**a**) baicalin or (**b**) baicalein for 24 h, the HIF-1α mRNA expression levels were determined by qRT-PCR analysis. GAPDH was used as a control to ensure an equal amount of loaded cDNA. Values are expressed as mean ± SD, ** *p* < 0.01, * *p* < 0.05 *vs.* control.

Baicalin and baicalein had different effects on HIF-1α mRNA expression in ovarian cancer cells and normal ovarian cells. Baicalin significantly increases HIF-1α mRNA expression in both cancer cells, while significantly decreases HIF-1α mRNA expression in IOSE-364 cells (Figure 4a). The reason why baicalin decreases HIF-1α mRNA expression but increases protein expression in normal cells is unclear. It might be because baicalin prevents HIF-1α protein degradation. More studies are needed to understand its mechanism. Baicalein significantly inhibited HIF-1α mRNA expression in CP70 and IOSE-364 cells at concentrations of 20-μM. But, when treatment concentration was 80-μM, HIF-1α mRNA expression increased rapidly to 233% in OVCAR-3 cells, 248% in CP70 cells and 256% in IOSE-364 cells (Figure 4b).

### 2.4. Effect of Baicalin and Baicalein on cMyc Protein Expression in OVCAR-3 and CP70 Cells

**Figure 5:**
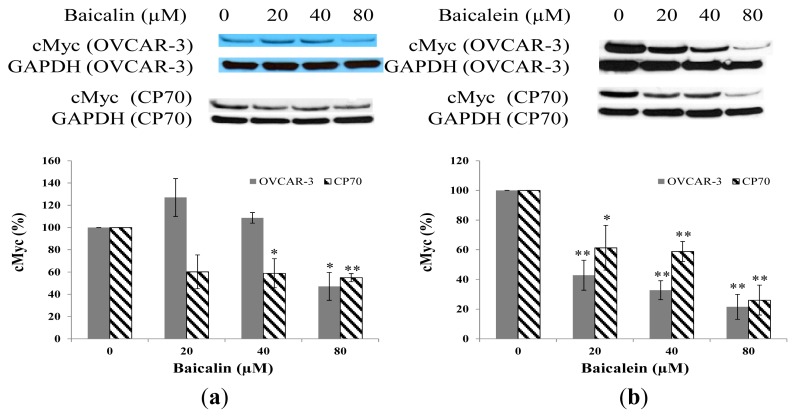
Effect of baicalin and baicalein on cMyc protein expression in OVCAR-3 and CP70 cells. Cells were treated with different concentrations of (**a**) baicalin or (**b**) baicalein for 24 h, the cMyc expression levels were determined by Western Blot analysis. GAPDH was used as a control to ensure an equal amount of loaded protein. Values are expressed as mean ± SD, ** *p* < 0.01, * *p* < 0.05 *vs.* control.

cMyc is reported to be an important oncogene which plays an important role in ovarian cancer growth. As shown in Figure 5a, baicalin decreased cMyc expression in CP70 cells. The inhibitory effect reached significant levels when the concentration of baicalin was 40 μM (*p* < 0.05) and 80 μM (*p* < 0.01). For OVCAR-3, baicalin decreased cMyc expression only when its concentration was 80 μM (*p* < 0.05). When the concentration of baicalin was 20 μM and 40 μM, baicalin had no significant effect on cMyc expression. However, baicalein significantly inhibited cMyc expression in both CP70 cells and OVCAR-3 cells (Figure 5b). Our results in ovarian cells agree with the findings in human leukemia cells that baicalin inhibits cell proliferation through down regulation of cMyc expression [30].

### 2.5. Effect of Baicalin and Baicalein on AKT Phosphorylation in OVCAR-3 and CP70 Cells

**Figure 6:**
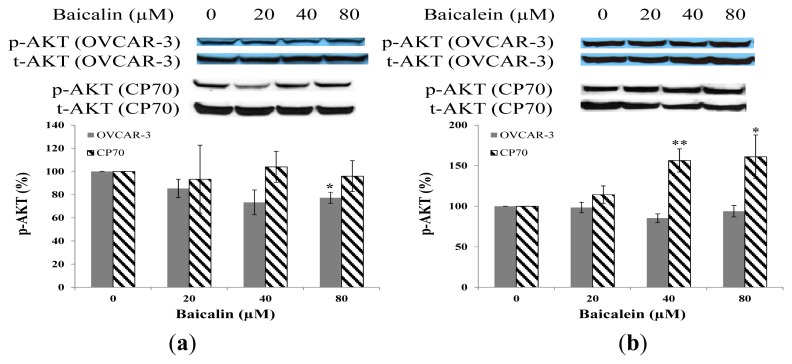
Effect of baicalin and baicalein on AKT phosphorylation in OVCAR-3 and CP70 cells. Cells were treated with different concentrations of (**a**) baicalin or (**b**) baicalein for 24 h, the P-AKT expression levels were determined by Western Blot analysis. Total-AKT was used as a control to ensure an equal amount of loaded protein. Values are expressed as mean ± SD, ** *p* < 0.01, * *p* < 0.05 *vs.* control.

Baicalin inhibited AKT phosphorylation OVCAR-3 cancer cells, but did not decrease AKT phosphorylation in CP70 cancer cells (Figure 6a). The AKT phosphorylation was not decreased by increasing concentrations of baicalein on both cancer cell lines (Figure 6b). Although it was found baicalein inhibited cell viability through inhibition of AKT phosphorylation in murine microglial cells [29], our results showed little effect of these compounds on AKT phosphorylation. Therefore, both compounds had little effect on the PI3K pathway since AKT phosphorylation is controlled by PI3K.

### 2.6. Effect of Baicalin and Baicalein on NFκB Protein Expression in OVCAR-3 and CP70 Cells

**Figure 7:**
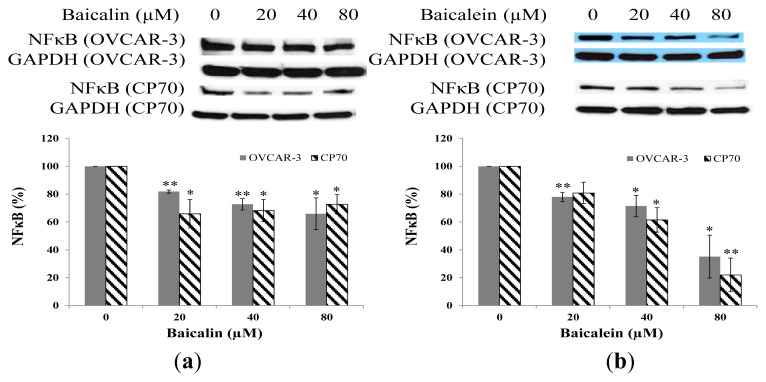
Effect of baicalin and baicalein on NFκB protein expression in OVCAR-3 and CP70 cells. Cells were treated with different concentrations of (**a**) baicalin or (**b**) baicalein for 24 h, the NFκB expression levels were determined by Western Blot analysis. GAPDH was used as a control to ensure an equal amount of loaded protein. Values are expressed as mean ± SD, ** *p* < 0.01, * *p* < 0.05 *vs.* control.

Due to it wide involvement in several pathways and broad regulation targets [31], the transcription factor NFκB is investigated here. Baicalin and baicalein had a certain impact on NFκB expression. The level of NFκB decreased with increases in baicalin and baicalein concentrations in OVCAR-3 and CP-70 cells (Figure 7a,b). The antitumor capacity of flavonoids can be largely attributed to their abilities to scavenge oxidative radicals, attenuate NF-κB activity, inhibit several genes important for regulation of the cell cycle, suppress COX-2 gene expression, and prevent viral infections [24].

### 2.7. Effect of Baicalin and Baicalein on PTEN Protein Expression in OVCAR-3 and CP70 Cells

**Figure 8:**
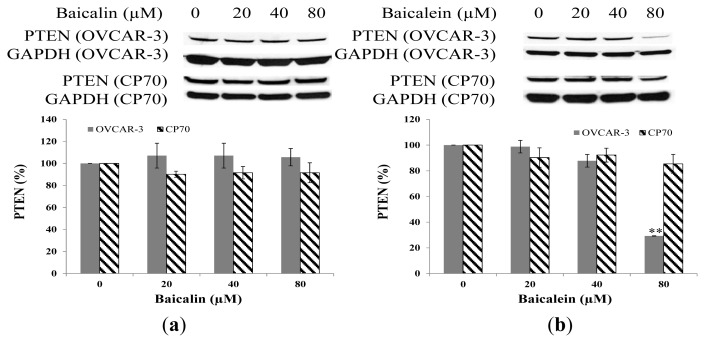
Effect of baicalin and baicalein on PTEN protein expression in OVCAR-3 and CP70 cells. Cells were treated with different concentrations of (**a**) baicalin or (**b**) baicalein for 24 h, the PTEN expression levels were determined by Western Blot analysis. GAPDH was used as a control to ensure an equal amount of loaded protein. Values are expressed as mean ± SD, ** *p* < 0.01, * *p* < 0.05 *vs.* control.

Figure 8a,b show the negligible effect on PTEN expression with increasing concentrations of baicalin and baicalein except for the effect of baicalein on OVAR-3 at 80 μM. PTEN is a tumor suppressor commonly mutated in many human cancers [32]. PTEN locates on 10q23.3, which encodes a 403-residue dual-specificity phosphatase that has protein phosphatase activity, and lipid phosphatase activity that antagonizes PI3K/AKT activity [33]. It seems that baicalin and baicalein had little effect on the PI3K pathway since both PTEN and AKT are important proteins in the pathway.

## 3. Experimental Section

### 3.1. Cell Culture and Treatment

Two human ovarian cancer cell lines, OVCAR-3 and A2780/CP70, and a normal ovarian cell line (IOSE-364) were maintained in RPMI 1640 medium supplemented with 10% US-qualified fetal bovine serum (Invitrogen, Grand Island, NY, USA) in a humidified incubator with 5% CO_2_ at 37 °C. The cells in logarithm proliferation period were used in the experiment. Baicalin and baicalein were dissolved in dimethyl sulfoxide (DMSO) to make stock solutions of 100 mM and equal amounts of DMSO were included in controls for every experiment.

### 3.2. Cell Viability

The effects of baicalin and baicalein on cell viability of ovarian cancer cells, OVCAR-3 and A2780/CP70 (CP70), and normal ovarian cells, IOSE-364, were colorimetrically determined with a “CellTiter 96 Aqueous One Solution Cell Proliferation Assay” kit from Promega (Madison, WI, USA). Cells (5000/well) were seeded into 96-well plates and incubated for 24 h before being treated with zero to 160 μg/mL baicalin and baicalein (containing 10% FBS RPMI 1640) in triplicates for another 24 h. After the medium was removed, the cells were washed with phosphate-buffered saline (PBS). Then 100 μL Aqueous one Reagent dilute solution (80 μL PBS + 20μL Aqueous one Reagent) was added to each well. Incubated at 37 °C for one and a half hours, cells were measured for OD values at 490 nm. Cell viability was expressed as a percentage of the control from three independent experiments.

### 3.3. ELISA for VEGF

Secreted VEGF protein levels were analyzed by sandwich ELISA with a Quantikine Human VEGF Immunoassay Kit from R&D Systems (Minneapolis, MN, USA) targeting VEGF in cell culture supernates. Cells (10,000/well) were seeded into 96-well plates and incubated for 16 h before treatment with baicalin and baicalein for 24 h. Culture supernates were collected for VEGF assay from a total of three independent experiments, each in triplicate, and were assayed. The total protein levels in the solution were used to normalize the VEGF concentration. The mean VEGF protein level from each duplicate was used for statistical analysis. A total of three independent experiments were carried out for statistical analysis.

### 3.4. Western Blot

Ovarian cancer cells and normal cells (10^6^) were seeded in 60-mm dishes and incubated for 16 h before treatment with baicalin and baicalein for 24 h. After washing with PBS, cells were harvested with 100 uL Mammalian Protein Extraction Reagent including 1 μL Halt Protease, 1 μL Phosphatase Inhibitor and 2 μL EDTA (M-PER, Pierce). Cells were then frozen at the temperature of −80 °C for 30 min, melted, and centrifuged at 12,000*g* at 4 °C for ten min, after which the aqueous phase was collected for measurement. Total protein levels were assayed with a BCA Protein Assay Kit (Pierce, Rockford, IL, USA), and lysates were separated by 10% SDS-PAGE and blotted into a nitrocellulose membrane with a Mini-Protean 3 System (Bio-Rad Laboratories, Hercules, CA, USA). For immunodetection, antibodies against HIF-1α (Prod# 610959, BD Biosciences, San Jose, CA, USA), NFκB (sc-114), PTEN (sc-7974), cMyc (sc-40), GAPDH (sc-47724, Santa Cruz Biotechnology, Santa Cruz, CA), p-AKT (9271L) and total AKT (9372S, Cell Signaling Technology, Beverly, MA, USA) were applied and signals visualized with X-ray film (Pierce Biotechnology, Rockford, IL, USA). Protein bands were quantitated with Quantity One software (Bio-Rad Laboratories, Hercules, CA, USA), normalized to corresponding GAPDH or total AKT bands, and expressed as percentages of the control. A total of three independent experiments were carried out for statistical analysis.

### 3.5. qRT-PCR

Cells (10^6^) were seeded in 60-mm dishes and incubated for 16 h before treatment with baicalin and baicelein for 24 h. After washing with PBS, cells were harvested in TRIzol reagent (Invitrogen, Grand Island, NY, USA) and stored in −80 °C until analysis. RNA samples were introduced to reverse transcription with AMV reverse transcriptase from Promega (Madison, WI, USA). cDNA was amplified by real-time PCR with RT^2^ SYBR Green qPCR Master Mix (SuperArray, Frederick, MD, USA) and a Chromo4t™ real-time detector coupled to a DNA Engine^®^ thermal cycler (Bio-Rad, Hercules, CA, USA). Primer sequences for HIF-1α is: FW: 5′-ATC CAT GTG ACC ATG AGG AAA TG-3′, RV: 5′-CTC GGC TAG TTA GGG TAC ACT T-3′; primer sequences for GAPDH is FW: 5′-CAT GAG AAG TAT GAC AAC AGC CT-3′, RV: 5′-AGT CCT TCC ACG ATA CCA AAG T-3′. The PCR program was set as follows: 95 °C 10 min (95 °C 10 s, 58 °C 45 s, 72 °C 20 s, 77 °C 1 s, read plate) × 40; 72 °C 5 min; 58 °C 1 min; melting curve (65 °C to 95 °C by 0.5 °C increments). A standard curve for each gene was generated from serial dilutions of PCR products to monitor amplification efficiency and to relatively quantify mRNA abundance. RNA samples without reverse transcription served as a non-reverse-transcription (−RT) control, and water served as a nontemplate control (NTC). Arbitrary units of each gene were derived from a corresponding standard curve, and mRNA abundance was normalized to GAPDH levels and expressed as percentages of control for statistical analysis. A total of three independent experiments were carried out for statistical analysis.

### 3.6. Statistical Analysis

Results were expressed as mean ± standard error of mean (SEM). The results were analyzed using one-way analysis of variance (ANOVA) and post hoc test (2-sided Dunnett’s *t*) to test both overall differences and specific differences between each treatment and control using SPSS version 16.0 (SPSS Inc., Chicago, IL, USA). Differences were considered significant at α = 0.05 level.

## 4. Conclusions

This research clearly showed that baicalin and baicalein have a significant impact on the viability and proliferation of ovarian cancer cells. On the other hand, both compounds showed less inhibition or increased expression on normal cells. Our results also showed that baicalin and baicalein significantly inhibited expression of cancer promoting genes including HIF-1α, cMyc, NFkB, and VEGF. Baicalein is more effective in inhibiting cancer cell proliferation and HIF-1 α, cMyc, NFkB and VEGF expression. It seems that baicalein inhibited cancer cell viability through the inhibition of cancer promoting genes expression including VEGF, HIF-1α, cMyc, and NFkB. The cancer fighting properties of baicalin and baicalein are still of interest and deserve further testing and research for possible application in chemoprevention and treatment of ovarian cancers.
